# Total polyphenols and antihyperglycemic activity of aqueous fruits extract of *Abelmoschus esculentus*: Modeling and optimization of extraction conditions

**DOI:** 10.1371/journal.pone.0250405

**Published:** 2021-04-16

**Authors:** Emanuel L. Peter, Prakash B. Nagendrappa, Clement Olusoji Ajayi, Crispin Duncan Sesaazi

**Affiliations:** 1 Department of Innovation, Technology Transfer and Commercialization, National Institute for Medical Research, Dar Es Salaam, Tanzania; 2 Centre for Local Health Traditions & Policy, The University of Trans-Disciplinary Health Sciences and Technology (TDU), Bengaluru, India; 3 Faculty of Medicine, Department of Pharmacy, Mbarara University of Science and Technology, Mbarara, Uganda; 4 Faculty of Medicine, Department of Pharmaceutical Sciences, Mbarara University of Science and Technology, Mbarara, Uganda; Institute for Biological Research, SERBIA

## Abstract

Aqueous fruits extract of *Abelmoschus esculentus* (L.) Moench (Malvaceae) has been used traditionally in several communities to alleviate elevated blood glucose levels. However, optimized extraction conditions have not been reported. Thus, this study determined the optimal extraction conditions for extracting polyphenols from *A*. *esculentus* fruits and evaluated antihyperglycemic activity *in vivo*. Extraction time, temperature, and solid-to-solvent ratio were optimized using Response Surface Methodology (RSM). Total polyphenols and flavonoids were quantified using the Folin-Ciocalteu and aluminium chloride colorimetric methods, respectively. The fingerprint and quantification of quercetin—a major flavonoid with an antihyperglycemic effect was done using the chromatographic method. The antihyperglycemic activity was determined in a high-fat diet-Streptozotocin rat model. The rats were assigned to five groups (n = 6): Group 1 and 2 were normal and diabetic control received distilled water 1 mL/100g; Treatment group 3 and 4 received standardized *A*. *esculentus* fruit extract (AEFE) at a dose of 100 and 200 mg/kg, respectively; Group 5 received 5 mg/kg glibenclamide. All treatments were given orally for 14 days. Measurements of fasting plasma glucose (FPG) and body weight were done weekly. The RSM quadratic model predicted total polyphenols of 22.16 mg GAE/g DW. At optimal conditions of a solid-to-solvent ratio of 5%, extraction time 1 h, and extraction temperature of 70°C, confirmation experiments yield 20.2 [95% CI; 16.7 to 27.6] mg GAE/g DW, implying the model successfully predicted total polyphenols. The extract HPLC fingerprint showed 13 characteristic peaks with 0.45 ± 0.02 μg/g DW of quercetin. Compared with diabetic control, the standardized AEFE reduced FPG level dose-dependently (*P* < 0.001) with an EC50 of 141.4 mg/kg. Together, at optimal extraction conditions, extract with a high content of total polyphenols and good antihyperglycemic activity can be obtained. Studies are needed to identify additional polyphenolic compounds and determine their antidiabetic effects.

## Introduction

*Abelmoschus esculentus* (L.) Moench (Malvaceae) is synonymously known as *Hibiscus esculentus* L., Okra, Okro, Ochro, lady’s finger, Gumbo, Gombo, Bamia (Kiswahili), Bamie, Bhandi, Quingombo, and Quiabo. It is an annual herbaceous plant, believed to have originated in Ethiopia is now almost cosmopolitan; cultivated in the tropical, subtropical, and warm temperate regions, particularly the Caribbean, South America, North America, Asia, rest of Africa, and eastern Mediterranea [1].

Traditionally, local communities have been using fruits of *A*. *esculentus* as food and herbal medicines for several ailments. Indians and Pakistanis are using fruits as curry [2]. Indonesians process fruits into soup or consume them as fresh vegetables [3]. The local community in India’s Jalgaon district chew raw fruits to aid digestion [4]; they also cook the fruits to manage gonorrhea, anemia & urethrorhea [5]. Similarly, Hachi and colleagues interviewed 1560 people in the Central Middle Atlas of Morrocco, including local healers, and found that the majority macerate 2 or 3 fruits in a glass of water overnight and take it during fasting for diabetes mellitus [6]. A recent study among traditional healers and individuals with diabetes mellitus in northern Tanzania found that the fruits of *A*. *esculentus* are among the most popular vegetable plant for this disease [7].

Numerous phytochemicals from young fruits and seeds of *A*. *esculentus* have been implicated for their traditional use in DM. These bioactive compounds were categorized as polyphenols, glycosides, alkaloids, polysaccharides, and volatile oils [8–10]. Polyphenol being the most prominent and ubiquitous class of plant secondary metabolites has several sub-classes that include flavonoids. The flavonoids exist either as free or bond to the sugar moiety. Researchers isolated myricetin -a free naturally occurring flavonoid from fruits of *A*. *esculentus* [11]. Similarly, quercetin flavonoid and its derivatives were also isolated from the fruits and their seeds [12–14].

The structural diversity of polyphenols contributed to their extensive bioactivities such as antioxidant, antidiabetic. Shen and colleagues showed that the fruits’ flavonoid fractions had a concentration-dependent α-glucosidase and α-amylase inhibitory effect [15]. In another study, researchers identified polyphenolic compounds -oligomeric proanthocyanidins from seeds, as active compounds responsible for α-glucosidase and α-amylase inhibitory activity [16]. Similarly, phenolic-rich extract and flavonoids isolated from *A*. *esculentus’s* seeds had excellent antioxidant activity *in vitro* [17] and, myricetin showed a dose-dependent decrease in the plasma glucose concentration and improved insulin sensitivity of obese Zucker rats [18]. The observed antidiabetic activity of pure polyphenolic compounds could imply that these compounds are partly responsible for the antidiabetic activity of crude extracts of *A*. *esculentus* [19]. The diverse mode of action of the phenol-rich extracts and pure polyphenols against DM could indicate a potential benefit to individuals with type 2 diabetes mellitus due to multiple organs’ involvement in its occurrence [20].

Extraction methods, extraction time, temperature, and type of solvents highly influence polyphenols’ extraction from plants, including *A*. *esculentus*. Previous studies have used water, methanol, ethanol, acetone, ethyl acetate, and their mixtures to extract polyphenols from flowers, fruits, leaves, seeds, and stem of *A*. *esculentus* [21,22]. Although these simple alcohols (methanol, ethanol), acetone, and ethyl acetate are environmentally friendly organic solvents [23], they are not adequately reflecting the traditional method of preparations which essentially involves using hot water maceration [6,7]. Also, the prolonged exposure to high temperature increases the hydrolysis and could quickly oxidize polyphenols leading to reduced extraction yield [24].

Thus, there is a need to establish optimal extraction conditions to maximize the extraction yield of polyphenols and enhance the extracts’ bioactive value. Therefore, this study aimed to determine the optimal extraction temperature, time, and solid-to-solvent ratio for aqueous extraction of polyphenols from young fruits of *A*. *esculentus* and to evaluate its antihyperglycemic property in the type 2 diabetes mellitus animal model.

## Materials and methods

### Materials

Gallic acid, aluminium chloride, sodium nitrate, sodium carbonate, and glibenclamide were of analytical grade purchased from Toronto research chemicals (Ontario, Canada), while HPLC grade; methanol, acetonitrile, trifluoroacetic acid, orthophosphoric acid, and quercetin were purchased from Sigma-Aldrich, Inc. Germany.

### Plant collection and extraction

Young fruits of *A*. *esculentus* were collected from a cultivated garden located along the northern bypass road at 0°34’53.0"S, 30°39’30.0"E, Mbarara, Uganda. Also, whole fresh aerial parts were collected from the same garden for taxonomical authentication. Mr. Protase Rwaburindori of the botany department at Makerere University identified and authenticated the plant specimen. A voucher specimen with accession number 50935 was deposited at the herbarium of the Makerere University. The fruits were cleaned with distilled water, chopped, and air-dried under shade to a moisture content of 8.2%. Later, the samples were crushed into a coarse powder using an electric grinder. The powder was weighed and stored in an airtight container under 4 ± 2°C until use.

### Design of experiment

We designed both single and multiple factors experiments. The Response Surface Methodology (RSM) with five-level three-factor central composite rotatable design (CCRD) was applied to determine the optimal combination of solid-to-liquid ratio, extraction time, and temperature that yield the maximum amount of total polyphenols. For this experiment, distilled water was used as an extraction solvent.

In a series of single-factor experiments (SFEs), each factor was assessed to give an experimental range. The effect of temperature on polyphenols’ extraction was determined by conducting triplicates *SFE* in a temperature range of 27, 40, 50, 60, 70, 80, and 90°C while other factors -time and solid-to-solvent ratio were fixed at 1 hour and 1:20 respectively. The temperature that gave the maximum yield of total polyphenols was used in the subsequent experiment. In the next experiment, the time in hours was varied; (0.5, 1, 2, 3, 5) while the solid-to-solvent ratio was maintained at 1:20. Finally, both the optimal time and temperature from previous experiments were used to establish the best solid-to-solvent ratio from the range of 2.5%, 3.3%, 5%, and 10%. Later, the RSM designed a multiple factor experiment (MFE) that had 20 experiments. The factors and levels were determined from the SFEs (Table 1).

**Table 1:** Coded and original values for independent variables used in the CCRD for optimization.

Factor	Name	Type	Actual		Coded		Mean ± SD
			Min.	Max.	Low	High	
**A**	Time (h)	Numeric	0.66	2.34	-1 ↔ 1	+1 ↔ 2	1.5 ± 0.42
**B**	Temp. (°C)	Numeric	43.18	76.82	-1 ↔ 50	+1 ↔ 70	60 ± 8.48
**C**	Ratio (%)	Numeric	1.65	5.85	-1 ↔ 2.5	+1 ↔ 5	3.75 ± 1.06

The 20 experiments were grouped into two blocks with total polyphenols content (TPC) as a response variable. The first block includes eight factorial points and four center points. The second block utilizes the six axial points of the design and two replicas at the center. The six replicas at the center were added to estimate the pure error sum of squares. The distance from the center (α) determines the extreme points, i.e., the lowest and highest values of the factors. With CCDR, the distance is 1.68179. The experiments were randomized to minimize unexplained variability in the observed response due to extraneous factors. Finally, confirmation experiments for the optimized extraction conditions for aqueous extraction of total polyphenols from fruits of *A*. *esculentus* were performed. The extract so obtained was subsequently standardized.

### Chemical standardization of aqueous fruit extract

#### Qualitative phytochemical screening

Qualitative phytochemical screening for alkaloids, carbohydrates, flavonoids, protein, phenols, saponins, steroids, and tannins was performed according to standard methods [25].

*Alkaloids*. Dragendrorff’s test determined the presence of alkaloids in the sample. About 1 mL of a sample solution was mixed with few drops of Dragendorff’s reagent. The formation of orange-yellow precipitate indicated the presence of alkaloids.

*Carbohydrates*. Benedict’s test determined the presence of carbohydrates in the sample. The sample solution was mixed with few drops of Benedict’s reagent (alkaline solution containing cupric citrate complex) and boiled in a water bath, forming a reddish-brown precipitate indicated a positive result for the presence of carbohydrate.

*Flavonoids*. The Shinoda test did detection of flavonoids in the sample. A small quantity of the sample was dissolved in 5 mL of 95% v/v ethanol. Then few drops of concentrated hydrochloric acid and 0.5 g magnesium turnings were added. The formation of pink or magenta color indicated the presence of flavonoids.

*Protein*. Firstly, the Biuret test was applied. About 1 mL of sample solution was treated with 10% sodium hydroxide solution, and two drops of 0.1% copper sulphate solution, the formation of violet/pink color indicated the presence of protein. Secondly, a Ninhydrin test was used. 2–3 drops of Ninhydrin reagent were added to the test tube containing 1 ml of the sample solution. A purple color formation indicates the presence of amino acids.

*Phenols*. The phenols were tested using Ferric Chloride and lead acetate. In the former test, about 1 mL of a sample solution was mixed with two drops of aqueous 0.1% Ferric Chloride. The formation of blue-black coloration indicated the presence of phenols. Whereas the later test involved about 1 mL of a sample solution mixed with three drops of lead acetate solution. The formation of precipitate indicated the presence of phenolic compounds.

*Saponins*. The foam test was used to determine saponins. According to this test, about 1 mL of sample solution was shaken with 5 ml of distilled water for 5 minutes. The formation of a stable, characteristic froth that lasts for at least 30 minutes indicated saponins’ presence.

*Steroids*. Salkowski test was used to determine steroids. To a 2 mL of the sample solution, about 5 mL of chloroform was added. Then, 1 mL of 98% concentrated sulphuric acid was added to the above mixture carefully along the test tube walls. The formation of a reddish-brown ring at the junction of two layers indicates the presence of steroids.

*Tannins*. The gelatin test was used for Tannins. A sample solution was treated with 1% w/v gelatin solution containing 10% sodium chloride. The formation of white precipitate determined the presence of gelatin.

#### Determination of total polyphenols content (TPC)

TPC of the extract was determined according to the Folin-Ciocalteu method [26]. About 1 mg/mL of the sample solution was oxidized with 2 mL of 10% v/v Follin-Ciocalteu reagent (FCR) and 2 mL of 7.5% sodium carbonate. The mixture was incubated for 40 minutes at 45°C, and absorbance was measured at 765 nm using a UV/VIS spectrophotometer (JENWAY, UK). The TPC was calculated as a Gallic acid equivalent based on a standard calibration curve.

#### Determination of total flavonoid content (TFC)

The extract’s total flavonoid content was determined according to the aluminium chloride colorimetric method [27]. Briefly, 1 mg/mL of crude extract was mixed with 4 mL of distilled water and 0.5 mL of 5% NaNO_2,_ then allowed to stand for 5 minutes. Later, 0.5 mL of 10% AlCl3 was added and allowed to stand for 6 minutes before adding 1 mL of 1 M NaOH and made the mixture 10 mL with distilled water. The mixture was allowed to stand for 15 minutes at room temperature, and absorbance was measured at 510 nm using a UV/VIS spectrophotometer (JENWAY, UK). The total flavonoid content was calculated as quercetin equivalent based on the standard calibration curve.

#### The HPLC fingerprint and quantification of quercetin

According to the previously developed and validated HPLC method, the extract’s fingerprint was determined [28]. The chromatographic system consisted of a Shimadzu LC-10AT equipped with an SPD-20A UV/VIS detector (Tokyo, Japan), communicator CBM-20A (Tokyo, Japan), and degassing unit DGU-20A_5R_ (USA) with an isocratic binary system of the mobile phase. Chromatographic separation was performed on a Lunar^®^ C_18_ column (5 μm; 250 x 4.6 mm; Phenomenex, USA) maintained at a temperature of 40°C in a Shimadzu column oven (CTO-20AC, Tokyo Japan). For chromatographic fingerprint, the mobile phase used was methanol: water: acetonitrile (60:30:10 v/v) containing 0.01% trifluoroacetic acid and a flow rate of 0.6 mL/min with a detection wavelength of 210. For quantification of quercetin, the mobile phase was methanol: 0.1% orthophosphoric acid water (60:40) and a flow rate of 1.0 mL/min with a detection wavelength of 370. In both fingerprint and quantification, the volume injected was 10 μL at a pressure of 2,596.17 psi. The sample was prepared in triplicate using methanol to a concentration of 1 mg/ml. Samples, mobile phase, and standard quercetin solution were filtered through 0.45μm membrane filters (EZ-Pak^®^, France) before loaded into the system.

### Assessment of antihyperglycemic activity

#### Animals

Healthy with no history of previous exposure, 16–19 weeks old male Wistar albino rats weighing 237–280 g were purchased from the animal research facility of the Department of Pharmacology, Mbarara University of Science and Technology. The animals were housed in clean cages under 20–25°C, a 12 h light/dark cycle, and were fed on standard rat pellets and water ad libitum for two weeks before the experiment.

#### Induction of type 2 diabetes mellitus (T2DM)

After high-fat diet (HFD) feeding for six weeks, animals fasted for 8 h followed by intraperitoneal injection of 30 mg/kg of freshly prepared Streptozotocin (STZ) in 0.1 M citrate buffer (PH 4.5). The HFD-low dose STZ produces a model of T2DM with features similar to those observed in patients with T2DM [29]. Five days later, the animals fasted for 8 h, and their FPG were measured. S1 Table shows a typical nutritional composition of the HFD. After induction, animals were assessed based on predetermined inclusion criteria; those with FPG >8.0 mmol/L, without co-morbid condition, and animals that were not on other treatment or previously exposed to other medicine were included in the study.

#### Experimental design and sample size determination

We used the Experimental Design Assistant (EDA) tool to generate a randomization sequence used to randomly assign 30 animals to five groups, with each group having six animals. The assignment to treatment groups was done by a technician who was not aware of the type of intervention each group assigned. However, during treatment and measurements, investigators were aware of the treatment groups since blinding was not possible given the significant difference in physical appearance between treatment and control interventions. The group size was determined with 0.80 power, 0.5 variabilities, a two-sided unpaired t-test at 0.05 significant level, and a unit change in FPG. This randomized in vivo study involved the following groups:

Group 1-Normal control; received distilled water (DW) 1 mL/100g
Group 2-Diabetic control received DW 1 mL/100 g
Group 3 and 4 were diabetic rats treated with AEFE at doses of 100 and 200 mg/kg, respectively
Group 5-Diabetic rats received Glibenclamide 5 mg/kg

The animals were individually identified to form an experimental unit. The interventions were given by oral gavage, and treatments continued for 14 days. S1 Fig shows a pictorial diagram of the experiment design. We also used Arrive checklist to ensure adequate design and implementation of the animal experiment (S1 Checklist)

#### Measurements

Primary outcome measure was fasting plasma glucose level (FPG): The FPG was measured from the rat’s tail blood using the Accu-Chek® Active (Roche, Mannheim, Germany) glucometer. The animals fasted for 8 h, and the blood was collected weekly (7 days’ interval). Secondary outcome measure was body weight: Animal weights in gram (g) were recorded using digital weighing balance. All measurements were performed at random. Finally, immediately after completion of the study period, the animals were euthanized by inhaling halothane in a closed chamber according to a previous protocol [30].

#### Data management and analysis

All animals with data on FPG and weight measured at baseline, days 7 and 14, were included in the analysis. Data were analysed using the Statistical Package for Social Sciences (IBM SPSS^®^) version 20.0 (Armonk, New York, USA). The mean and standard deviation of triplicates were calculated. Then, data were summarized into bar graphs. A one-way analysis of variance (ANOVA) was applied for hypothesis testing. Since homogeneity of variance was not assumed (Levene statistic was statistically significant, p-value < 0.001), we reported Welch’s p-value as a robust test of equality of means and later used Gomes-Howell multiple comparison tests for multiple comparisons mean between treatment groups. The p-value of less than 0.05 was considered statistically significant. The effective concentration (EC50) value representing the concentration of extract that exerts 50% of its maximal response was determined by regression analysis, using a Quest Graph™ EC50 Calculator [31].

Further analysis of experimental data was done using Design-Expert software version 12 (StatEase, Minneapolis, USA). Then data were fitted in a higher-order quadratic polynomial model as given in Eq 1.

$$Y=\beta_{0}+\sum_{j=1}^{k}\beta_{j}X_{j}+\sum_{j=1}^{k}\beta_{jj}X_{j}^{2}+\sum \sum_{i<j}^{k}\beta_{j}X_{i}X_{j}+\sum \sum_{i\tilde{\neq }j}^{k}\beta_{ij}X_{i}^{2}X_{j}$$ (1)

*Y* represents the response variable (TPC), and X_i_ and X_j_ (*i*≠*j*) are independent factors. The *β*_0_, *β*_*j*_, *β*_*jj*_ and *β*_*ij*_ are regression coefficients for the model intercept, linear, quadratic, and interaction terms. The regression parameters obtained from the multiple regression models quantify the impact of the three independent factors on the extraction efficiency, taking into account their interaction. The model’s adequacy was determined by assessing the *p*-value of the lack of fit, coefficient of determination (*R*^2^), and ANOVA F-value. The three-dimensional response surface graph was plotted for optimal TPC level against two independent factors.

#### Ethical considerations

Mbarara University of science and technology research ethical committee (MUST-REC) approved the study protocol (MUST-REC 19/01-19). Also, the protocol was approved by the Uganda National Council for Science and Technology (UNCST) with research registration number NS119ES. The experiments were carried out according to the guidelines for the care and use of laboratory animals of the National academy of sciences.

## Results

### Effect of single independent factors

#### Temperature

**Fig 1:**
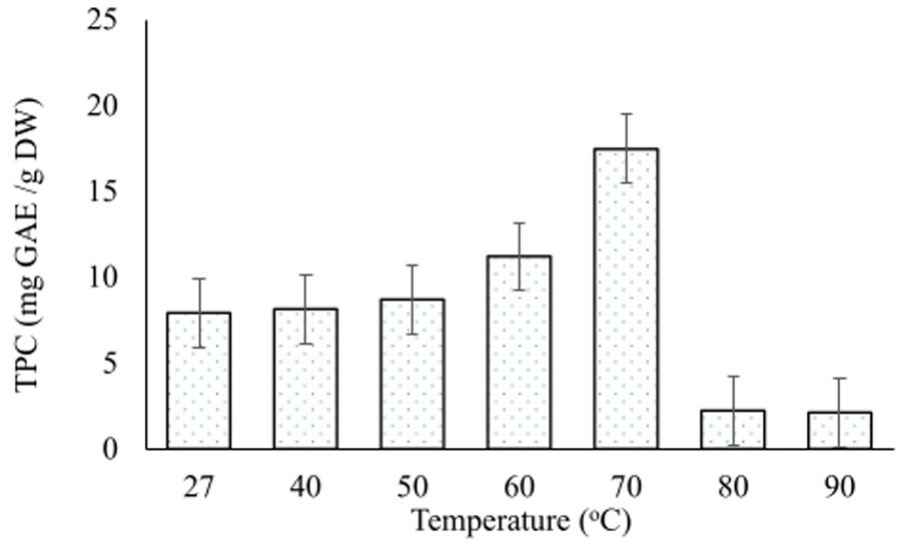
Effect of temperature on extraction of total polyphenols.

Extraction of polyphenols increased with the increase in temperature and reached a peak (17.4 mg GAE/g DW) at 70°C. Beyond this temperature, TPC sharply decreased to a minimum of 2.1 mg GAE/g DW (Fig 1).

#### Time

**Fig 2:**
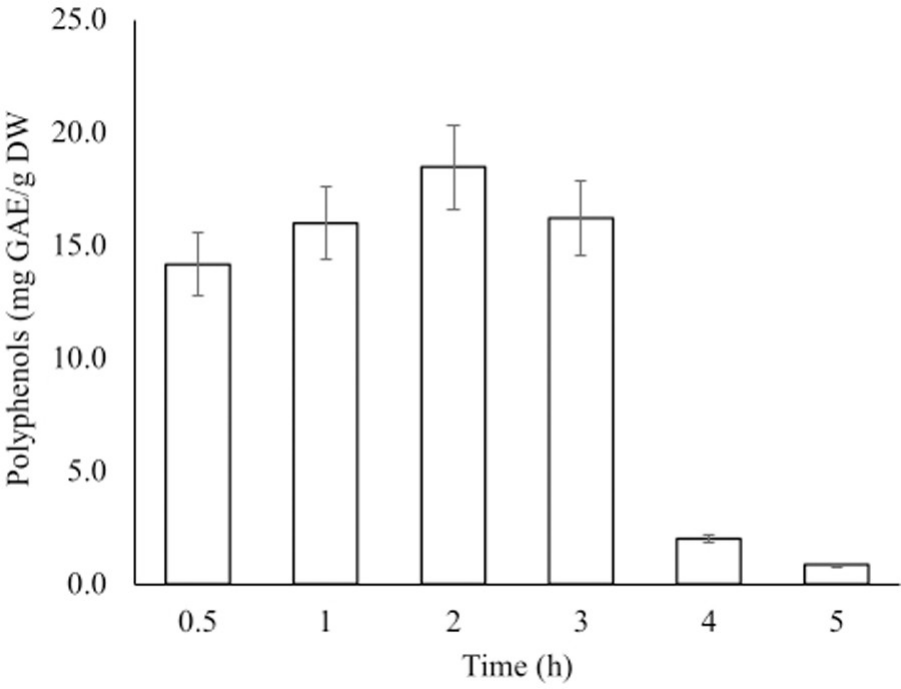
Effect of contact time on the extraction of total polyphenols from fruits of *A*. *esculentus*.

The effects of extraction time on TPC are shown in Fig 2. The yield of TPC increased with the increase in time up to 2 hours that gave the maximum yield of 18 mg GAE/g DW. The increase was then followed by a gradual decrease to a minimum of 0.9 mg GAE/g DW.

#### Solid-liquid ratio

**Fig 3:**
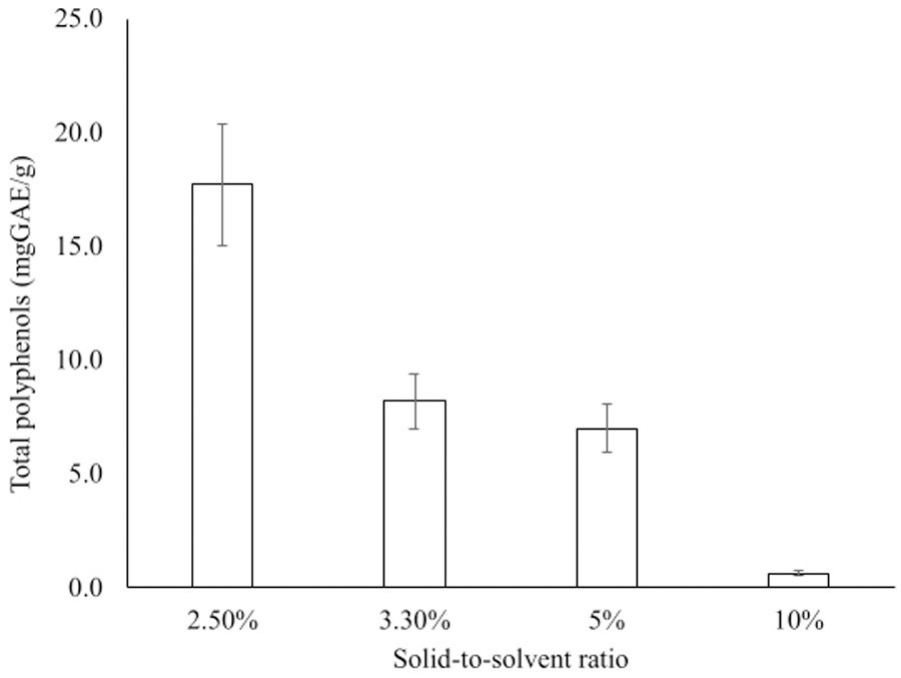
Effect of solid-to-solvent ratio on the extraction of total polyphenols from fruits of *A*. *esculentus*.

The maximum amount of TPC (17.7 mg GAE/g DW) was obtained at 2.5% (Fig 3). The yield was then gradually decreased with a decrease in the extraction solvent.

### Multiple factor experiments

The findings of the central composite design with the three factors are shown in Table 2. In this experiment, TPC was calculated based on the calibration curve (*R*^*2*^ = 0.9923) and expressed as milligram of Gallic acid equivalent/gram of dry weight of the sample (mg GAE/g DW).

**Table 2:** The experimental factors and the actual value.

Run	Block	Time (h)	Temperature (°C)	Solvent-solid-ratio (%)	TPC (mg GAE/g DW
**1**	Week 1	1	50	5	14.54
**3**	Week 1	1.5	60	3.75	16.60
**3**	Week 1	1.5	60	3.75	15.90
**4**	Week 1	1.5	60	3.75	15.96
**5**	Week 1	1	50	2.5	13.20
**6**	Week 1	2	70	5	19.21
**7**	Week 1	2	70	2.5	21.29
**8**	Week 1	1.5	60	3.75	16.10
**9**	Week 1	1	70	5	17.25
**10**	Week 1	2	50	2.5	12.46
**11**	Week 1	2	50	5	10.54
**12**	Week 1	1	70	2.5	18.00
**13**	Week 2	0.66	60	3.75	16.67
**14**	Week 2	1.5	60	1.65	10.08
**15**	Week 2	1.5	60	5.85	15.25
**16**	Week 2	1.5	60	3.75	13.46
**17**	Week 2	1.5	77	3.75	17.29
**18**	Week 2	1.5	43	3.75	12.10
**19**	Week 2	2.34	60	3.75	14.63
**20**	Week 2	1.5	60	3.75	15.82

The results of ANOVA for the quadratic model are shown in Table 3. The Model F-value indicated that the model was significant (P = 0.0079). This means there is only a 0.79% chance that an F-value this large could occur due to noise. The *P*-values less than 0.05 imply that model terms were significant, which means B, C, AB, AC, and B2 were significant model terms for TPC extraction. The terms; A, BC, A^2^, and C^2^, although not significant, could not be removed from the model as they support the hierarchy of the model function.

**Table 3:** Analysis of variance for a fitted quadratic model.

Source	Sum of Squares	df	Mean Square	F-value	p-value
**Block**	24.49	1	24.49		
**Model**	299.84	9	33.32	4.74	0.0079*
**A-Time**	1.28	1	1.28	0.2207	0.6496
**B-Temp**	55.69	1	55.69	9.59	0.0128*
**C-Ratio**	38.12	1	38.12	6.57	0.0306*
**AB**	34.03	1	34.03	5.86	0.0385*
**AC**	91.69	1	91.69	15.79	0.0032*
**BC**	0.1250	1	0.1250	0.0215	0.8866
**A²**	7.45	1	7.45	1.28	0.2867
**B²**	58.39	1	58.39	10.76	0.0113*
**C²**	10.76	1	10.76	1.85	0.2064
**Residual**	52.25	9	5.81		
**Lack of Fit**	39.94	5	7.99	2.60	0.1882
**Pure Error**	12.31	4	3.08		
**Cor Total**	376.58	19			

The lack of Fit of 2.60 was not statistically significant relative to the pure error (P = 0.1882). It means there is an 18.82% chance that a lack of fit F-value this large could occur due to noise. The *R*^2^ = 0.8516 indicates factors explain the response variable (TPC) sufficiently. The adeq precision, which is a measure of signal-to-noise ratio, was 10.45. This value indicates an adequate signal, and therefore our model can be used to navigate design space. The final equation in terms of coded factors is indicated in Eq (2).

$$\text{TPC}=14.75–0.3063*\text{A}+2.02*\text{B}+1.67*\text{C}–2.06*\text{AB}–3.39*\text{AC}+0.1250*\text{BC}+0.7191*{\text{A}}^{2}–2.01*{\text{B}}^{2}–0.8645*{\text{C}}^{2}$$ (2)

Eq (2) can be used to make predictions about the yield of TPC for given levels of each factor. By comparing the factor coefficients, this equation could be used for identifying the relative impact of the factors on the extraction of TPC from fruits of *A*. *esculentus*. Finally, the quadratic model so fitted predicted mean TPC as 22.163 mg GAE/g DW.

**Fig 4:**
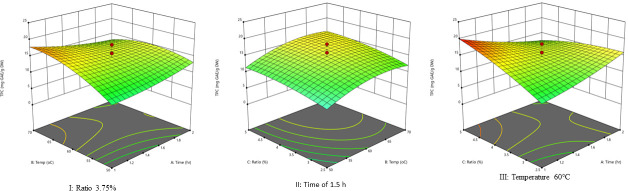
Response surface plots. The response surface plot **I** show the interactions of extraction temperature and time at a fixed solvent-to-solid ratio on total polyphenols, the response surface plot **II** indicates the interaction effects of solid-to-solvent ratios and temperature at a constant time on extraction efficiency of total polyphenols, and the plot **III** indicate the interaction of time and solvent-to-solid ratios at a fixed temperature at 60°C.

The 3D graphs show the effect the two factors have on TPC extraction when one factor is maintained constant (Fig 4). Fig 4 (I) presents the relationship of TPC, extraction temperature, and time at a fixed solvent-to-solid ratio. The TPC amount increases with the increase in temperature until it reached the highest level of about 17 mg GAE/g DW, and the extraction efficiency of TPC increases with time to a maximum of 2 h. Similarly, Fig 4 (II) shows the effect of varying solid-to-solvent ratios and temperature on extraction efficiency for TPC. Accordingly, when the ratio was kept constant at 3.75, the increased temperature gradually increased TPC, reached the highest amount, and gradually decreased. Similarly, when the temperature was kept constant at 60°C, the decrease in solvent gradually increase TPC.

### Optimization of extraction conditions

Optimal extraction conditions that meet the criteria for maximizing the yield of total polyphenols from fruits of *A*. *esculentus* were selected from numerical optimization. These conditions were: extraction time of 1 h, temperature 70°C, and the solid-to-solvent ratio of 5% w/v at a desirability level of 0.61. Confirmation experiments were performed in triplicates. The results showed a mean of 20.2 [95% CI; 16.7 to 27.6] mg GAE/g DW, which implies that the model successfully predicted the extraction of total polyphenols from fruits of *A*. *esculentus*. The extract obtained was termed AEFE and subsequently used for chemical standardization and bioactivity assay.

### Chemical standardization of AEFE

#### Qualitative phytochemical screening

The AEFE was found to have alkaloids, glycosides, reducing sugars, phenol, tannins, amino acids, saponins, steroids, and flavonoids. Table 4 illustrates the reagents used and the intensity of reactions.

**Table 4:** Preliminary phytochemical screening.

Test	Reagents/test	Observations	*A*. *esculentus*
**Alkaloid**	Dragendrof’s	Orange precipitate	+++
**Carbohydrates**	Benedict’s	Reddish-brown	+
**Flavonoids**	Shinoda test	Pink colour	++
**Protein**	0.1% Ninhydrin	Purple colour	++
**Phenols**	5% FeCl_3_ lead acetate solution	Green colour Formation of precipitate	+
**Saponins**	Distilled water	Foamy	+++
**Steroids**	Chloroform and conc. H_2_SO_4_	Formation of the brown ring at the junction of the two ring	++
**Tannins**	1% w/v gelatin	White precipitate	+

+ traces present; ++ moderate present; +++ adequate.

#### Total flavonoid, HPLC fingerprint, and quantification of quercetin

**Fig 5:**
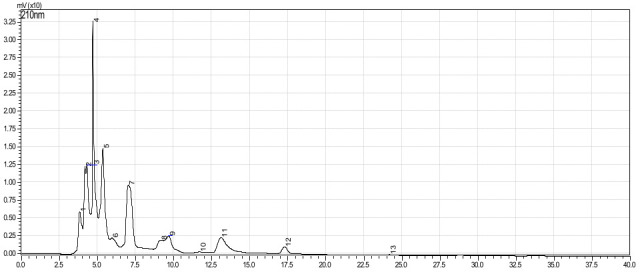
The HPLC fingerprint of aqueous extract of *A*. *esculentus*.

Based on the calibration curve (R^2^ = 0.9903), the total flavonoid content of the AEFE was estimated as 12.4 mg QE/100g. The HPLC chromatogram of AEFE shown 13 characteristic peaks (Fig 5) and Table 5. This can be used for identification purposes and monitor the quality of crude extract. The HPLC quantitative method quantified the amount of quercetin as 0.45 μg/g DW based on the calibration curve (R^2^ = 0.9997).

**Table 5:** The HPLC peaks of *A*. *esculentus* fruits extract.

Peak #	RT	Area	Height	% area	% height
**1**	3.794	201032	1196	7.511	7.626
**2**	3.963	182217	17960	6.808	12.233
**3**	4.164	293826	27584	10.977	18.788
**4**	4.544	829269	37788	30.982	25.738
**5**	4.968	657958	25133	24.581	17.119
**6**	5.523	182944	14381	6.835	9.795
**7**	6.059	30332	1880	1.133	1.28
**8**	6.467	74535	2830	2.785	1.928
**9**	7.497	1274	132	0.048	0.09
**10**	8.066	213665	7665	7.983	5.221
**11**	19.81	5744	137	0.215	0.093
**12**	23.544	2731	90	0.102	0.061
**13**	49.665	1118	41	0.042	0.028
**Total**		2676645	146818	100	100

RT = retention time.

### The standardized AEFE lower elevated plasma glucose level

The oral administration of the AEFE at doses 100 mg/kg and 200 mg/kg significantly reduced fasting plasma glucose at day 7 and day 14 (*P* < 0.001). The reduction of fasting plasma glucose was more (6.0 ± 0.61) mmol/L in a group given 200 mg/kg compared to that received 100 mg/kg (6.5 ± 0.37) mmol/L. However, the observed difference in response between the two doses was not statistically different (*P* = 0.467). There was no statistically significant difference in mean fasting plasma glucose at day 14 between the groups receiving polyphenolic-rich extract and standard Glibenclamide (*P* > 0.05). Table 6 illustrates the various comparisons. Using the sigmoid dose-response equation, the EC50 of the extract was calculated as 141.4 mg/kg.

**Table 6:** Effect of standardized AEFE on fasting plasma glucose.

Group	Dose (mg/kg)	FPG (mmol/L)		
		Day 1	Day 7	Day 14
**1) Normal control**	DW (1 mL/100g)	5.5 ± 0.34	5.5 ± 0.23 ^b^	6.2 ± 0.33 ^b^
**2) Diabetic control**	DW (1 mL/100g)	11.1 ± 0.87 ^a^	16.1 ± 0.90	18.1 ± 1.21
**3) AEFE**	100	10.6 ± 1.04 ^a^	7.8 ± 0.50 ^b^^,^^c^	6.5 ± 0.15 ^b^^,^^c^
**4) AEFE**	200	12.6 ± 1.02 ^a^	7.9 ± 0.35 ^b^	6.0 ± 0.23 ^b^
**5) Glibenclamide**	5	10.7 ± 0.79 ^a^	8.0 ± 0.39 ^b^^,^ ^d^	6.4 ± 0.25 ^b^^,^ ^d^

AEFE: *Abelmoschues esculentus* fruit extract; HFD-STZ: High-fat diet-Streptozotocin; FPG: Fasting plasma glucose; DW: Distilled water; n = 6 for each groups; Data are expressed in mean ± SEM;

^*a*^*P* < 0.05 for treatment groups compared with normal control;

^*b*^*P* < 0.001 for AEFE 100 and 200 mg/kg, glibenclamide 5 mg/kg compared with diabetic control;

^*c*^*P* > 0.05 for AEFE 200 mg/kg compared with AEFE 100 mg/kg;

^*d*^*P* > 0.05 for AEFE 100 and 200 mg/kg compared with glibenclamide 5 mg/kg.

### The effect of standardized AEFE on body weight

According to Table 7, normal control and the three treatment groups (100, 200, 5 mg/kg) had their weight increased compared to their average baseline weight. However, a comparison of mean weights among these groups at day 14 shows no statistically significant difference (P = 0.578). Conversely, the diabetic control group’s mean body weight on day 14 was lesser than the baseline weight (Table 7). The decrease in body weight could be due to the effect of diseases.

**Table 7:** Effect AEFE on body weight of HFD-STZ induced diabetic rats.

Group	Dose (mg/kg)	Weight (g)		
		Day 1	Day 7	Day 14
**1) Normal control**	DW (1 mL/100g)	261 ± 10.22	262 ± 10.46	262 ± 10.46
**2) Diabetic control**	DW (1 mL/100g)	273 ± 7.44	269 ± 17.34	268 ± 15.35
**3) AEFE**	100	273 ± 11.65	281 ± 13.68	290 ± 14.09
**4) AEFE**	200	268 ± 7.14	269 ± 6.25	274 ± 7.93
**5) Glibenclamide**	5	237 ± 17.77	251 ± 15.82	265 ± 15.71

AEFE: *Abelmoschus esculentus* fruit extract; HFD-STZ: High-fat diet-Streptozotocin; DW: Distilled water; n = 6 for each group; Data are expressed in mean ± SEM; ANOVA P = 0.578.

## Discussion

Extraction is an early step to obtain bioactive compounds such as polyphenols and flavonoids from plant materials. The extraction temperature, solvent, the particle size of the plant materials, the solvent-to-solid ratio, and the extraction duration affect extraction efficiency [32]. Our findings suggest that interactions between extraction time, temperature, and solid-to-solvent ratio significantly influence the aqueous extraction of total polyphenols from fruits of *A*. *esculentus*. The best extraction efficiency could be achieved at a temperature of 70°C, an extraction time of 1 hour, and the solid-to-solvent ratio of 5% w/v in the simple maceration method. These findings have important implications for the broader domain of traditional use of the crude extract of *A*. *esculentus* to manage diabetes mellitus. More often than not, such traditional practices involve preparing the extract from raw or dried fruits with water using a traditional maceration technique. Maceration method remains the popular extraction method due to its inherent advantages such as its simplicity, most miniature experimental setup, less energy and time consuming, and provide high polyphenols yield [33–35]. Our results that maceration effectively extracts total polyphenols from fruits under optimal extraction conditions agree with the previous study [33].

Previous studies established that solvent type affects the amount and rate of total polyphenols extracted from plant materials due to properties such as polarity differences [36]. Unlike the present study that used water extraction solvent, earlier studies extracted total polyphenols from *A*. *esculentus* using methanol, ethanol, and their varied combination with water [21,22]. Although such solvents resulted in a good yield of total polyphenols [37], the use of organic solvent could not provide a more practical application to the traditional use of *A*. *esculentus*, thus, rendering previous study findings; of limited use at the community level.

The RSM quadratic model successfully predicted total polyphenols. The verification experiment confirmed the predicted polyphenols as 20.2 mg GAE/g DW. The use of RSM to optimize polyphenols’ extraction has increasingly become a preferable statistical technique in recent years [38,39]. The growing popularity of RSM in the extraction of bioactive compounds could be attributed to its ability to assess the interaction of the several independent variables on the response and optimize the process parameters to a reasonable level at a few experiments [39].

Contrary to our findings, Liao et al., 2012 reported a lower yield of 12.4 ± 0.12 mg GAE/g DW. Their experiment used 80% methanol at 50°C for 30 minutes in an ultrasonic cleaner [22]. Indeed, differences in the solvent used, extraction time, and temperature could have contributed to the observed differences in the amount of total polyphenols. Besides, the lack of optimization of extraction factors hampered their study’s ability to assess the potential interaction effects among factors on polyphenols’ yield. In another study, Geng et al., 2015 reported a high TPC yield of 40.77 ± 0.83 mg GAE/g DW from *A*. *esculentus* flower using optimized extraction time of 2.5 h with an ethanol concentration of 59.16% at a temperature of 73.91°C and the liquid-solid ratio of 20 mL/g [38]. Since the temperature and the solid-to-solvent ratio are almost similar to those optimized in our study, it follows then that the difference in solvents could explain the difference in TPC yield as reported in other studies [40,41].

Herbal extracts have multiple chemical constituents, some of which therapeutic values are yet to be ascertained [42]. This situation has made chromatographic and spectrophotometric methods indispensable for qualitative, semi-quantitative, and quantifying chemical marker compounds for extract standardization [43]. Also, the chromatographic fingerprint has gained recognition as an essential tool in herbal extract standardization [43]. The WHO and US FDA encourage fingerprint use to ensure herbal extract quality consistency (WHO, USA FDA). In realization of such a need to standardize extract, to ensure quality and reproducibility of findings, our study performed additional phytochemical analysis. The qualitative phytochemical analysis results found that the extract (AEFE) had alkaloids, carbohydrates, phenol, tannins, proteins, saponins, steroids, and flavonoids. Also, the HPLC fingerprint indicated about 13 characteristic peaks. This phytochemical and fingerprint information could be considered tools for monitoring the quality of extract and identification purposes. Also, our study identified quercetin as a chemical marker compound with a known antidiabetic activity [44,45]. Accurate identification of this flavonoid compound and its occurrence in appreciable quantity in AEFE further assure potential antidiabetic activity of the AEFE. Besides, it could also be used to monitor the quality of the extract [46].

Our findings that standardized aqueous crude extract of *A*. *esculentus* significantly reduce FPG in animals are similar to those reported in another study [47]. Conversely, the aqueous extract had no significant effect on body weight. These results validate the traditional use of the aqueous crude extract to manage elevated blood glucose among individuals with type 2 diabetes mellitus at the community level. They also support the need to consider appropriate temperature, extraction time, and solid-to-solvent ratio for maximum yield of total polyphenols from the fruits.

## Conclusion

The RSM quadratic model successfully predicted the aqueous extraction conditions for maximum yield of polyphenols from young fruits of *A*. *esculentus*. The standardized polyphenolic extract demonstrated an antihyperglycemic activity in animals. These results underscore the traditional use of aqueous extract to ameliorate hyperglycemia in people with type 2 diabetes mellitus. Furthermore, nutraceutical and herbal industries could consider these optimized extraction parameters for polyphenol-rich product development. Future researches are needed to identify additional polyphenolic compounds from the extract and assess their antidiabetic effects. Also, mechanistic studies to understand how the standardized extract reduces elevated plasma glucose levels are worth further research.

## Supporting information

S1 Checklist ARRIVE checklist. (PDF)

S1 Fig Pictorial presentation of experimental design. (TIF)

S1 Table Nutrition composition of a normal diet and high-fat diet (HFD). (PDF)
