# Supplementary figures and images for: Total polyphenols and antihyperglycemic activity of aqueous fruits extract of Abelmoschus esculentus: Modeling and optimization of extraction conditions
Source: PLoS One. 2021 Apr 16;16(4):e0250405. doi: 10.1371/journal.pone.0250405 (PMC8051779; doi:10.1371/journal.pone.0250405)

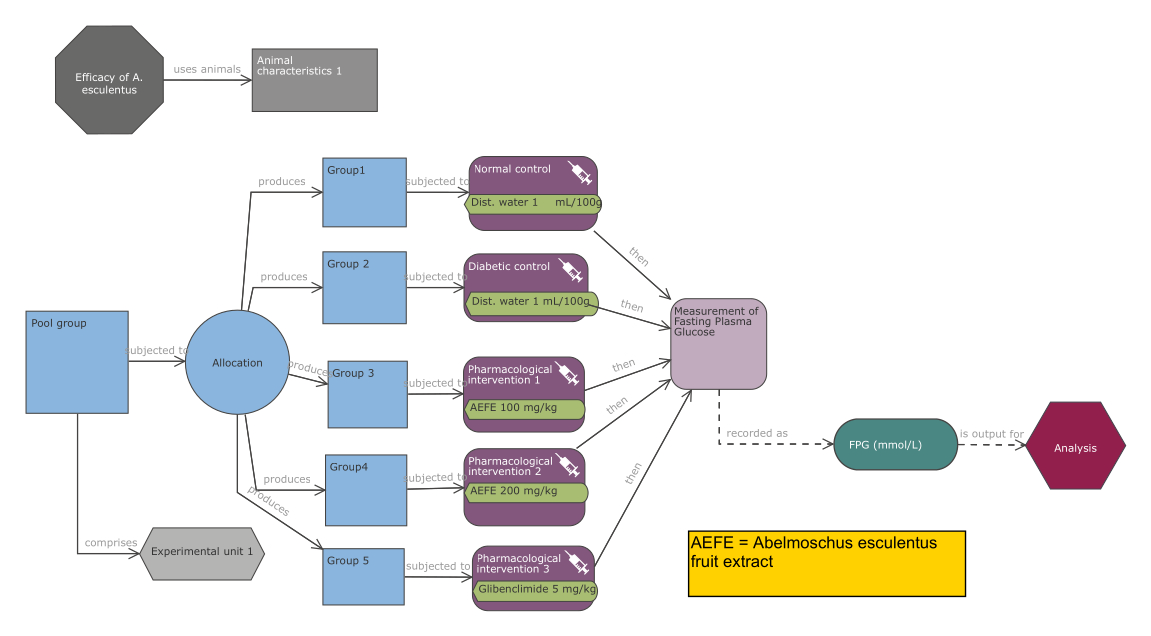

Supplement: S1 Fig — (TIF) [file pone.0250405.s002.tif]
