# Supplementary material for: Total polyphenols and antihyperglycemic activity of aqueous fruits extract of Abelmoschus esculentus: Modeling and optimization of extraction conditions
Source: PLoS One. 2021 Apr 16;16(4):e0250405. doi: 10.1371/journal.pone.0250405 (PMC8051779; doi:10.1371/journal.pone.0250405)
Supplement: S1 Table — (PDF) [file pone.0250405.s003.pdf]

Table 1. Nutrition composition of normal diet and high-fat diet (HFD)

Nutrients source	Normal diet		HFD	
	Quantity [g]	Calories [Kcal/g]	Quantity [g]	Calories [Kcal/g]
Casein	200	800	200	800
Lysine	2	8	2	8
Methionine	1	4	1	4
Threonine	0.5	2	0.5	2
Corn starch	412	1648	179	716
Sucrose	397	1588	180	720
Corn oil	20	180	20	180
Lard	30	270	230	2070
Minerals mix	10	0	10	0
Vitamin mix	10	0	10	0
Calcium carbonate	5.5	0	5.5	0
Cellulose	35	0	35	0
Total energy	1069	4,500	838	4,500

HFD = High-fat diet
